# Dispersive Liquid-Liquid Microextraction Combined with Ultrahigh Performance Liquid Chromatography/Tandem Mass Spectrometry for Determination of Organophosphate Esters in Aqueous Samples

**DOI:** 10.1155/2014/162465

**Published:** 2014-01-29

**Authors:** Haiying Luo, Yanping Xian, Xindong Guo, Donghui Luo, Yuluan Wu, Yujing Lu, Bao Yang

**Affiliations:** ^1^Guangzhou Quality Supervision and Testing Institute, Guangzhou 510110, China; ^2^School of Chemical Engineering and Light Industry, Guangdong University of Technology, Guangzhou 510110, China; ^3^Key Laboratory of Plant Resources Conservation and Sustainable Utilization, South China Botanical Garden, Chinese Academy of Sciences, Guangzhou 510650, China

## Abstract

A new technique was established to identify eight organophosphate esters (OPEs) in this work. It utilised dispersive liquid-liquid microextraction in combination with ultrahigh performance liquid chromatography/tandem mass spectrometry. The type and volume of extraction solvents, dispersion agent, and amount of NaCl were optimized. The target analytes were detected in the range of 1.0–200 **µ**g/L with correlation coefficients ranging from 0.9982 to 0.9998, and the detection limits of the analytes were ranged from 0.02 to 0.07 *µ*g/L (*S*/*N* = 3). The feasibility of this method was demonstrated by identifying OPEs in aqueous samples that exhibited spiked recoveries, which ranged between 48.7% and 58.3% for triethyl phosphate (TEP) as well as between 85.9% and 113% for the other OPEs. The precision was ranged from 3.2% to 9.3% (*n* = 6), and the interprecision was ranged from 2.6% to 12.3% (*n* = 5). Only 2 of the 12 selected samples were tested to be positive for OPEs, and the total concentrations of OPEs in them were 1.1 and 1.6 *µ*g/L, respectively. This method was confirmed to be simple, fast, and accurate for identifying OPEs in aqueous samples.

## 1. Introduction

Organophosphate esters (OPEs), a type of organophosphorus flame retardant, are well known for their antiflaming property. Since brominated diphenyl ether flame retardants have been gradually restricted worldwide, OPEs are used as an alternative in textile, chemical, electronic, and other industries. Given that OPEs are mixed into products rather than chemical reaction, they can easily diffuse into the surrounding environment, where most OPEs are fairly persistent [1–8]. Most OPEs are poisonous or otherwise harmful to human beings. For instance, trichloroethyl phosphate (TCEP) has been found to be carcinogenic. Tri(chloropropyl) phosphate (TCPP) exhibits potential carcinogenicity. Tributyl phosphate (TBP) and triphenyl phosphate (TPhP) are neurotoxins, while TPhP inhibits hormone levels [3–8]. In addition, chlorinated phosphate esters such as TCEP and TCPP are hard to be degraded naturally. As a result, the detection of OPEs has become a new direction for organic pollutant test [1–9].

Recently, various studies regarding the analysis of OPEs have been carried out, and OPEs were found in the atmosphere, water, soil, and *Larus argentatus* eggs [1–18]. To date, researches worldwide have mostly focused on textile matrices [19–21], but few studies were published concerning water mediums. Only Wang et al. [17] and Yan et al. [8] reported the existence of OPEs in water in Songhua River and Tai Lake, respectively. Present pretreatment techniques for the determination of OPEs from water samples are based on solid-phase extraction (SPE), which requires large sample volumes and is time consuming [8, 13–17]. Current analytical methods for the determination of OPEs mainly include gas chromatography equipped with nitrogen phosphorus detector (GC-NPD) [21], gas chromatography/mass spectrometry (GC-MS) [8, 20], and ultrahigh performance liquid chromatography/tandem mass spectrometry (UPLC-MS/MS) [13–18]. Amongst, the GC-NPD method [22] was demonstrated to have poor stability and be difficult to achieve the confirmation of target analytes. GC-MS suffers from unfavorable fragmentations [22] and is with lower selectivity when compared with MS/MS, while UPLC-MS/MS under the multiple reaction monitoring (MRM) mode can acquire satisfactory selectivity and sensitivity that is suitable for the trace detection of OPEs in aqueous samples [7, 9, 15, 17, 18].

**Figure 1:**
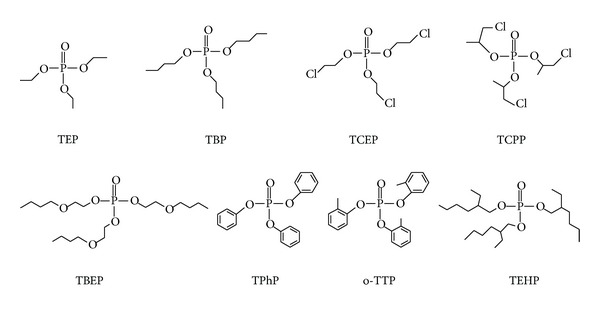
Structures of eight organophosphate esters (OPEs).

Dispersive liquid-liquid microextraction based on the solidification of floating organic drop (DLLME-SFO) technique combines the procedures of sampling, extraction, and concentration into one step. This method is characterized by the simplicity of operation, low cost, and high recovery. It also shows great potential for trace analysis [23, 24]. In this study, the DLLME-SFO technique was applied for the first time to the detection of OPEs, and UPLC-MS/MS was combined to develop a reliable method for the determination of OPEs in aqueous samples. Eight OPEs (Figure 1) were separated and detected within 4 min. The method is rapid, precise, and sensitive, and it can be used for the analysis of aqueous samples. Furthermore, its potential can be extended to investigate the pollution by OPEs in other fields.

## 2. Experimental

### 2.1. Instruments, Reagents, and Materials

ACQUITY ultrahigh performance liquid chromatography (UPLC, Waters, USA) was interfaced to a triple quadrupole tandem mass spectrometry (Xevo TQ MS, Waters, USA). The centrifuge, vortex mixer, and Milli-Q Gradient system were obtained from LD5-2A (Jingli, Beijing, China), MS3 (IKA, Germany), and Millipore (Bedford, USA), respectively.

Triethyl phosphate (TEP, ≥99.9%), TBP (≥99.5%), TPhP (≥99.5%), TCEP (≥98.5%), tris(2-ethylhexyl)phosphate (TEHP, ≥98.0%), tributoxyethyl phosphate (TBEP, ≥95.8%), TCPP (≥67.3%), tritolyl phosphate (o-TTP, ≥97.0%), and tributyl phosphate-d27 (TBP-d27, ≥99.7%) were purchased from Dr. Ehrenstorfer, Germany. HPLC-grade methanol and acetonitrile were acquired from Merck (Darmstadt, Germany). HPLC-grade formic acid was purchased from Fluka (Buchs, Switzerland). Undecanol (98%) was purchased from Aladdin, and analytical-grade NaCl (Guangzhou Chemical Reagent Factory, China) was obtained from Guangzhou. Ultrapure water (18.2 MΩ) was obtained from a Milli-Q system (Millipore, Bedford, USA).

Individual stock solutions of eight OPEs and an internal standard (TBP-d27) were prepared using methanol after being dissolved in acetone. Mixed stock solution containing all analytes was prepared in methanol at concentration of 1 mg/mL. Internal standard TBP-d27 solution was also diluted with methanol to the same concentration and then stored at 4°C. The mixed standard solutions were obtained by being diluted with undecanol/methanol (4 : 6, v/v) to obtain the concentrations of interest for the OPEs and achieve a concentration of TBP-d27 (20 *μ*g/L).

The water samples included 10 types of bottled drinking water (purchased from a local market), tap water samples from our lab, and one sample from the Pearl River.

### 2.2. Sample Preparation

A 10 mL glass centrifuge tube, 10 *μ*L of the 1.0 mg/L TBP-d27 internal standard solution, and 2.0 g NaCl were dissolved in 8.0 mL of the water samples by vortex shaking. 300 *μ*L of methanol were added into the solutions, which were mixed by vortexing, before adding 400 *μ*L of undecanol. After vortexing for 2 min and centrifuging at 3000 rpm (1610 g) for 5 min, the centrifuge tube was then stored in 0°C ice water for 5 min. When undecanol solidified, the solution from the tube was discarded, and the solidified undecanol was then trapped on the filter paper and transferred into a 1 mL dry graduated tube. After reconstituting with methanol and diluting to a volume of 0.5 mL, the solution was transferred into a sample tube for the UPLC-MS/MS test.

### 2.3. UPLC-MS/MS Analysis

The separation of the OPEs was accomplished using a UPLC system equipped with a Phenomenex Kinetex PFP column (50 × 3.5 mm, 2.6 *μ*m). The mobile phase was 0.1% formic acid aqueous solution (A) and acetonitrile (B), at a flow rate of 0.3 mL/min. The gradient was set as follows: 0.0–0.5 min (60% A), 0.5–2.0 min (60–20% A), 2.0–5.0 min (20% A), 5.0–5.1 min (20–60% A), and 5.1–7.0 min (60% A). The injection volume was 5 *μ*L, and the column temperature was 30°C.

A triple-quadrupole mass spectrometer was interfaced to the UPLC for the determination of the OPEs. Electrospray ionization (ESI) was performed in the positive-ion mode with a capillary voltage of 0.5 kV, a source temperature of 150°C, a desolvation temperature of 500°C, a nebulizer gas flow of 50 L/h, and a drying gas flow of 1000 L/h. Argon pressure in the collision cell was maintained at 0.15 mL/min. The quantification of all compounds was performed in MRM mode. Individual MS/MS results for the eight analytes and internal standard are shown in Table 1. The residence time of each ion pair was 0.006 s.

## 3. Results and Discussion

### 3.1. Extraction Solvent and Volume

The polarity of the eight OPEs had a wide range of log⁡⁡*K*_ow_ values from 0.80 (TEP) to 9.49 (TEHP) [5, 8]. The density of the extraction solvent used for DLLME-SFO should be less than that of water, so that it can suspend on the surface of the solution. Meanwhile, the extractant should have a melting point near room temperature, which can be frozen using an ice bath and separated easily from water. Consequently, only a small amount of solvent is required to reconstitute for analysis [23, 24]. Considering that liquid-phase separation was employed in this study, different types of extraction solvents with melting points around room temperature, such as undecanol, dodecanol, and tetradecyl alcohol, were compared.

**Figure 2:**
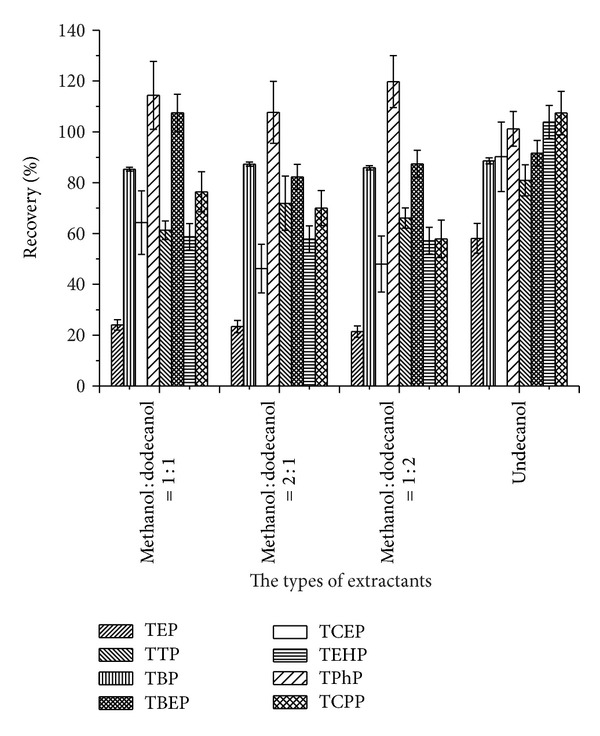
Optimization of extractants.

Since tetradecyl alcohol possesses a slightly higher melting point than the other solvents, when dissolved in methanol and added into aqueous samples, it was immediately solidified to small particles and suspended on the solution surface. A solid block could not be formed by freezing. Hence, it could not meet the requirement of extraction and sample injection for analysis. Given that undecanol and dodecanol can fulfill the requirements for DLLME-SFO, undecanol and different volume ratios of methanol/dodecanol (dodecanol solidified at room temperature was used after being heated in a water bath and dissolved in methanol) including 1 : 1, 2 : 1, and 1 : 2 were considered. The recovery values of the eight OPEs using the different extraction solvents are shown in Figure 2. The results revealed that undecanol showed better extraction efficiency. Seven of the OPE samples had favorable recoveries between 85% and 117%. However, TEP exhibited a relatively lower recovery of 53% (attributed to the particularly low log⁡⁡*K*_ow_). Therefore, undecanol was chosen as the extraction solvent. Since the melting point of undecanol was relatively low, which would result in fusion loss during phase separation, the extraction temperature should not be high, and the operation should be fast.

**Figure 3:**
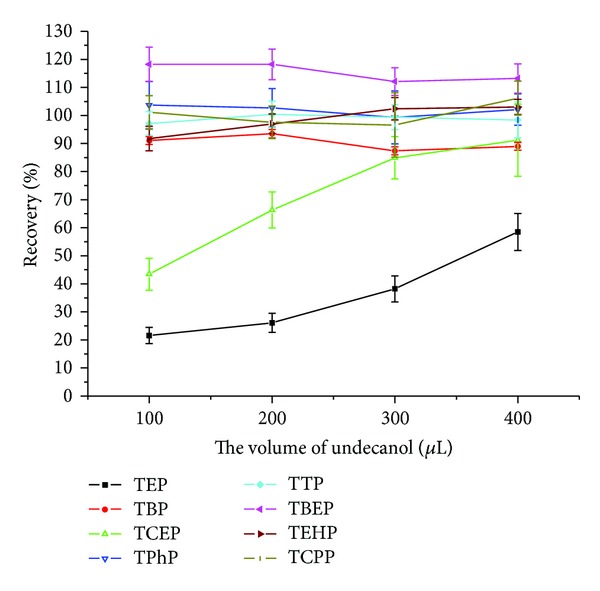
Optimization of extractant volume.

During extraction, an appropriate solvent volume was essential for efficient extraction. Too little extraction solvent made it difficult to efficiently extract all the analytes, while an increase in the extraction solvent volume led to an increased extraction yield, but a lower limit of detection, because the analyte enrichment was impaired. In the present work, extraction solvent volumes of 100, 200, 300, and 400 *μ*L were investigated with respect to their extraction efficiencies. As shown in Figure 3, with the increase in solvent volume, the extraction efficiency of TEP was markedly enhanced, while that of the other OPEs presented low solvent volume sensitivities. Because of the high water solubility of TEP, only larger volumes of extraction solvents could give sufficient distribution of TEP in the organic phase. Thus, 400 *μ*L of extraction solvent was selected in this study with the prerequisite of maintaining the detection sensitivity.

**Figure 4:**
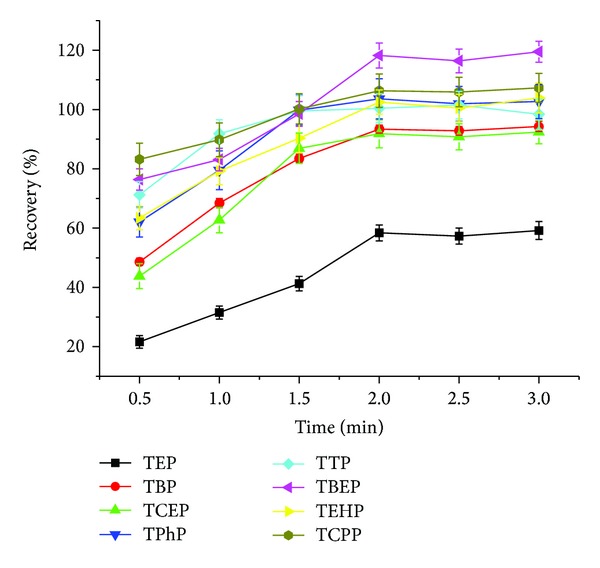
Optimization of vortexing time.

High-speed vortexing was employed to ensure that the solvents form extremely small liquid drops, so that a sufficient contact area with the sample could be achieved. The recoveries of all the analytes indicated that extraction reached equilibrium in a short time (2 min), as shown in Figure 4.

### 3.2. Dispersive Solvent and Volume

**Figure 5:**
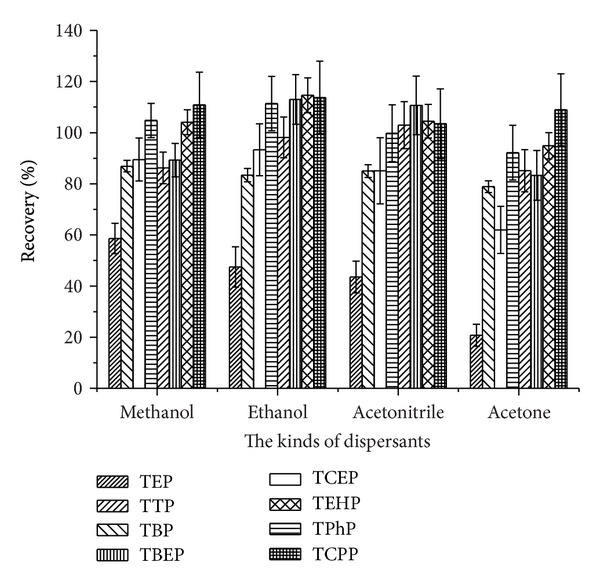
Optimization of dispersant.

The polarity of the dispersive solvent should be between water and the extractant, so that the dispersant can be completely miscible with the water and extraction solvent. In this study, methanol, ethanol, acetonitrile, and acetone were used as dispersants to evaluate their dispersion and extraction effects. The results presented in Figure 5 indicated that these dispersants exhibited favorable dispersion effects, while methanol exhibited the best extraction efficiency and was chosen as the dispersive solvent in this study.

**Figure 6:**
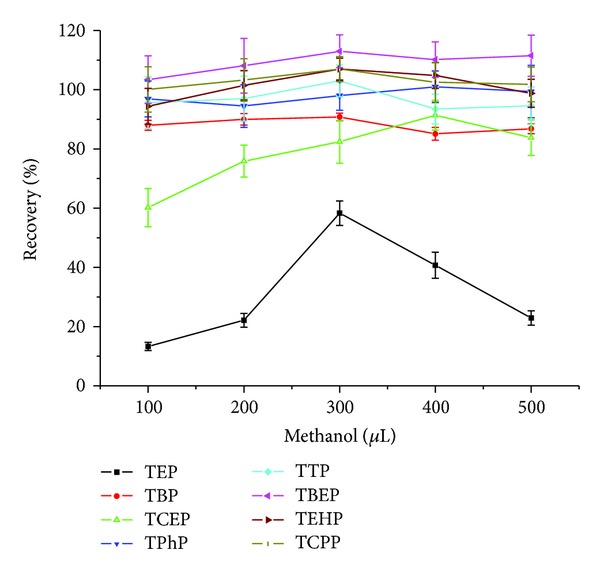
Optimization of dispersant volume.

The volume of the dispersant is an important factor for extraction efficiency, where too low and too high volumes lead to a decreased efficiency. An emulsion will not be formed if the dispersant is absent, while an excess of dispersant will result in the reduction in the partition coefficient of analytes in the extractant. Different volumes of 100, 200, 300, 400, and 500 *μ*L of methanol were assessed, and the resulting extraction efficiencies are shown in Figure 6. These results indicated that TEP, TBP, TEHP, and TTP had the highest extraction efficiencies with 300 *μ*L of methanol while TCEP showed the highest value at 400 *μ*L of methanol, and the efficiencies of the other three analytes demonstrated no apparent dependence on the dispersant volume. Given that the extraction efficiency of TCEP at 300 *μ*L of methanol did not show a sharp decline compared with 400 *μ*L, consequently, 300 *μ*L of methanol was selected for the present study.

### 3.3. Influence of the Salting-Out Effect

**Figure 7:**
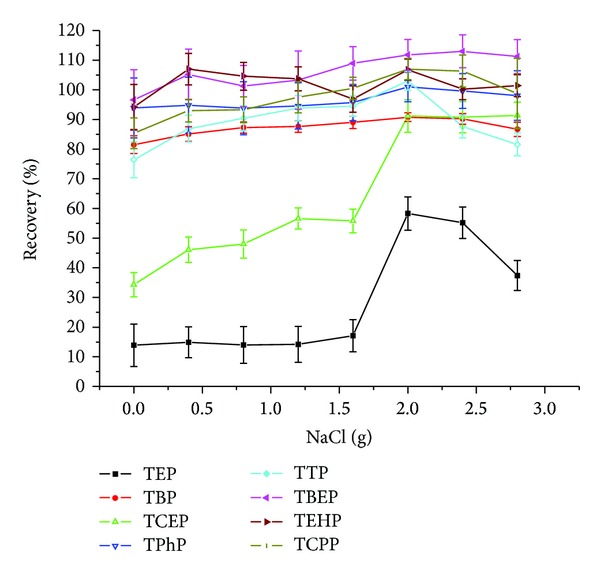
Optimization of amount of NaCl.

The addition of salt could enhance the ionic strength and reduce the solubility of the analytes and organic extractant in the aqueous phase. Thus, salt addition is beneficial for improving extraction efficiency. Optimization was performed by adding various amounts of NaCl (0, 0.4, 0.8, 1.2, 1.6, 2.0, 2.4, and 2.8 g) to the system, and the results are shown in Figure 7. For all of the target compounds, different levels of improvement in the extraction yield were obtained by increasing the amount of NaCl. With the addition of 2.0 g NaCl, TEP was found to have a significant increase in extraction efficiency, suggesting that the solubility of TEP in water could effectively decrease at this ionic strength. Meanwhile, the other OPEs exhibited satisfactory extraction yields under these conditions. Hence, 2.0 g of NaCl was selected as the optimum amount.

### 3.4. Optimization of Measurement Conditions

A relatively high abundance of quasimolecular ion peaks [M+H]^+^ was observed in the ESI collision mode for eight OPEs, where three hydrogen atoms of phosphoric acid are replaced by substituents (Figure 1). The characteristic parent ions selected for Q3 scanning were optimized. The qualitative and quantitative ion pairs of the eight OPEs were selected according to the MS fragmentation mechanism [14], and the collision energy was optimized correspondingly to obtain the highest intensity of each characteristic fragment ion. The MS parameters of the eight OPEs are shown in Table 1.

**Figure 8:**
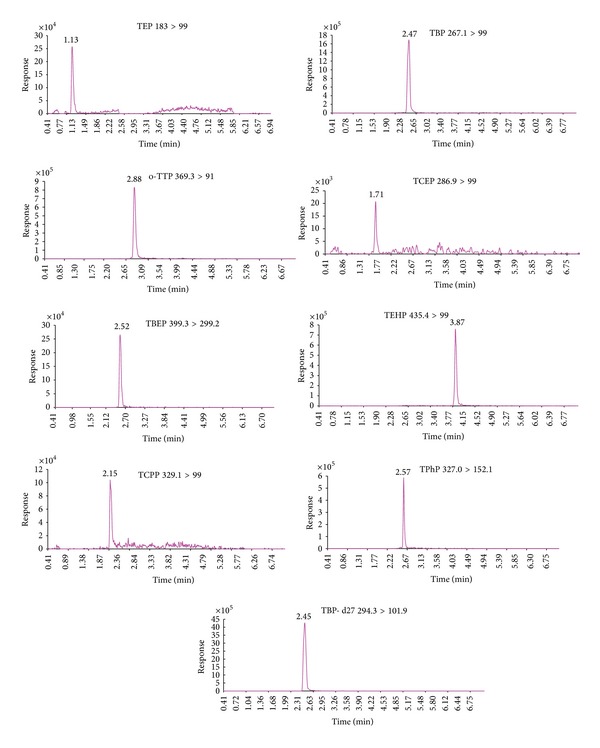
Selected ion chromatograms of the mixed standard solution of OPEs (5.0 *μ*g/L) and internal standard TBP-d27 (20.0 *μ*g/L).

Formic acid was added to the mobile phase to enhance the ionization efficiency by providing the essential proton source for the target analytes. The elution separation of all the analytes was accomplished within 4 min. The selected ion chromatograms of the mixed OPE standards and TBP-d27 internal standard are shown in Figure 8.

### 3.5. Linearity and LOD

A series of mixed OPE standard solutions was prepared. The series concentrations of TBP, o-TTP, TBEP, TPhP, and TEHP were set at 1.0, 2.0, 10.0, 50.0, 100, and 200 *μ*g/L, those of TEP and TCPP were 2.0, 5.0, 20.0, 50.0, 100, and 200 *μ*g/L, and those of TCEP were 3.0, 5.0, 20.0, 50.0, 100, and 200 *μ*g/L. Each of the mixed standard solutions was spiked with 20.0 *μ*g/L of TBP-d27 internal standard. Under optimized UPLC-MS/MS conditions, the linear equations were obtained by setting the specific values between the peak areas of each target compound and internal standard as ordinate (*y*) but the corresponding mass concentrations as abscissa (*x*).

The LOD was calculated by analyzing the spiked aqueous sample that underwent pretreatment and yielded a signal-to-noise ratio of 3 (*S*/*N* = 3). The equations of linear correlation, correlation coefficient, and the LOD of the target analytes are shown in Table 2, which indicates that the OPEs presented favorable linearity with the correlation coefficient larger than 0.998 within the corresponding concentration range and LODs of 0.02–0.07 *μ*g/L.

### 3.6. Recoveries and Reproducibility

Negative water samples at three spiked levels of analyte with 10 *μ*L 1.0 mg/L TBP-d27 were used to test the recoveries of analytes according to the proposed method with six identical samples tested at each concentration. The results are shown in Table 3. It indicated that the recoveries of seven OPEs were satisfactory with values in the range of 85.9%–113%, with TEP exhibiting significantly lower recoveries of 48.7%–58.3%. Relative standard diversities (RSDs, *n* = 6) of 3.2%–9.3% were observed, while the intraday variability was 3.6%–12.3%.

### 3.7. Application to Practical Samples

**Figure 9:**
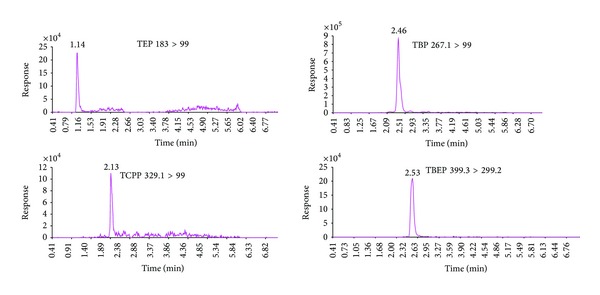
Ion chromatograms of OPEs in the Pearl River water samples.

The analytical method described here was utilized to determine OPE levels in different aqueous samples, including 10 types of bottled drinking water purchased from the local market, tap water from our lab, and water from the Pearl River. No OPEs were detected in any of the bottled drinking water. TEP (1.5 *μ*g/L) and TEHP (0.1 *μ*g/L) were found in the tap water, which may arise from contaminant leaching from the plastic pipe connected to the water outlet. In the case of the water sample from the Pearl River, TEP, TCPP, TBP, and TBEP were found to be present at concentrations of 0.2, 0.4, 0.2, and 0.3 *μ*g/L, respectively. The selected ion chromatograms of the targets in the Pearl River water samples are presented in Figure 9.

### 3.8. Compared with Other Methods

In this work, ultrahigh performance liquid chromatography/tandem mass spectrometry was confirmed to be effective in chemical quantitation and qualitation [25]. The methods for OPEs extraction from aqueous samples include liquid-liquid extraction (LLE), solid-phase extraction (SPE), solid-phase microextraction (SPME), membrane-assisted solvent extraction (MASE), and dispersive liquid-liquid microextraction (DLLME). Although LLE and SPE are the most commonly used, they are time and solvent consuming, with low extraction efficiency for TCEP and TEHP (31% for TCEP and 24%–51.8% for TEHP) [22, 26]. SPME that commonly use PDMS-DVB fiber [26, 27] showed low extraction efficiency for TEHP (about 26.7%–64.8%) and poor repeatability. Ionic liquid-based sol-gel fiber that was self-constructed laboratorially by Gao and coworkers [22] for headspace solid-phase microextraction, which enhanced the extraction efficiency of TEHP and repeatability, is of cockamamie manipulation and high technology requirement and is hard to be promoted. MASE is also quite time-consuming, while DLLME [28] exhibited unsatisfactory recovery for TEHP (about 40%). In spite of the recovery of TEP in the present research which is not in a favorable level (48.7%–58.3), it is stable, in an acceptable range, and can fulfill the requirement of detection. Satisfyingly, high recoveries and precision of the other seven OPEs can be obtained by using the present method. The comparisons of the different extraction methods are shown in Table 4. As could be seen that most of the researches focus on TnBP and TEHP, except this study, none of the other researches are involved in the detection of TEP. Compared with the other researches listed in Table 4, although the present study showed relatively high LOD, it is the result of the least volume of sampling and enrichment.

## 4. Conclusions

A method for identifying eight different organophosphate esters, TEP, TBP, TCEP, TBEP, TCPP, TTP, TPhP and TEHP, in aqueous samples has been established by the combination of DLLME-SFO and UPLC-MS/MS. The type and volume of the extraction solvent, dispersion agent, and the amount of added NaCl were optimized. The linearity, detection limit, recovery of the analyte, and precision of the method were investigated. The results indicated that this method was effective and reliable for the qualitative and quantitative analysis of OPEs in aqueous samples. It had advantages of simple pretreatment, high precision, and high recovery. This work also extended the application of DLLME-SFO technique and provided useful information for research regarding environmental and dietary pollution by OPEs.

## Figures and Tables

**Table 1:** MS parameters of OPE compounds and TBP-d27 internal standard.

Compounds	Precursor ion (*m*/*z*)	Daughter ion (*m*/*z*)	Cone voltage (V)	Collision energy (eV)
TEP	183.0	99.0^a^	18	18
		127.0	18	12
TCEP	286.9	99.0^a^	22	26
		161.1	22	16
TCPP	329.1	99.0^a^	16	22
		175.0	16	14
TBP	267.1	99.0^a^	18	18
		155.1	18	10
TBEP	399.3	299.2^a^	26	12
		199.1	26	16
TPhP	327.0	152.1^a^	40	32
		77.0	40	36
o-TTP	369.3	91.0^a^	46	32
		165.0	46	42
TEHP	435.4	99.0^a^	16	14
		323.0	16	6
TBP-d27	294.3	101.9	20	18

^a^Transitions for quantification.

**Table 2:** Equations of linear correlation, correlation coefficients, and method detection limits (LOD) of OPEs.

Analyte	Linear regression	Correlation coefficient	LOD (µg/L)
TEP	*y* = 0.0122*x* − 0.0148	0.9993	0.07
TCEP	*y* = 0.0006*x* − 0.0011	0.9990	0.07
TCPP	*y* = 0.0032*x* − 0.0027	0.9992	0.04
TBP	*y* = 0.0647*x* − 0.0094	0.9995	0.02
TBEP	*y* = 0.0122*x* − 0.0293	0.9982	0.02
TPhP	*y* = 0.0097*x* − 0.0003	0.9998	0.03
o-TTP	*y* = 0.0292*x* − 0.0252	0.9991	0.02
TEHP	*y* = 0.0219*x* − 0.0148	0.9993	0.02

*y*: peak area of analytes/peak area of TBP-d27; *x*: mass concentration, µg/L.

**Table 3:** Recoveries and precisions of OPEs.

Analyte	Spiked (µg/L)	Recovery (%)	RSD (%, *n* = 6)	Interday variability (%, *n* = 5)
TEP	0.3, 0.6, 3.0	48.7, 52.8, 58.3	6.9, 6.2, 6.9	6.8, 5.6, 5.3
TCEP	0.3, 0.6, 3.0	85.9, 86.8, 91.4	9.3, 8.1, 5.9	12.3, 10.2, 5.8
TCPP	0.2, 0.4, 2.0	107, 94.2, 95.6	7.3, 5.3, 4.1	7.0, 5.7, 4.3
TBP	0.1, 0.2, 1.0	90.8, 86.4, 89.4	3.9, 3.0, 4.3	2.6, 2.8, 3.0
TBEP	0.1, 0.2, 1.0	113, 95.6, 102	7.4, 3.2, 3.8	7.5, 5.6, 3.9
TPhP	0.1, 0.2, 1.0	101, 97.2, 95.8	6.5, 5.7, 4.5	4.6, 4.5, 3.8
o-TTP	0.1, 0.2, 1.0	103, 94.6, 98.8	6.9, 3.7, 3.2	3.2, 4.5, 6.4
TEHP	0.1, 0.2, 1.0	107, 99.8, 102	5.0, 3.0, 3.6	4.3, 3.3, 2.7

**Table 4:** Extraction effects for OPEs of different methods.

Extraction method	Detection	Targets	Recovery (%)	RSD (%)	LOD (ng/L)	Ref.
LLE (25 mL, DCM)	LC-MS/MS	TnBP, TEHP	80–94	1.9–12	TnBP: 11 TEHP: 7.2	[9]

SPE (HLB) (>60 min)	LC-MS/MS	TnBP, TEHP	TnBP: 65–90 TEHP: 50–70	TnBP: 1–11 TEHP: 1–16	TnBP: 20 TEHP: 38 (LOQ)	[14]

SPE (HLB) (2000 mL sample)	GC-NPD	TiBP, TnBP, TCEP, TCPP, TDCP, TBEP, TPhP, TEHP, and TPPO	24–109	2.1–16.7	5–10	[25]

SPME (PDMS-DVB fiber, 20 mL, 30 min)	GC-NPD	Ditto	26.7–119.2	5.3–64.8	15–25 (LOQ)	[25]

MA-HS-SPME (PDMS-DVB fiber, 20 mL, 5 min)	GC-MS (SIM)	TnBP, TEHP	86–106	6–15	TnBP: 0.2 TEHP: 1.5	[26]

SPME (IL-based sol-gel fiber, 10 mL, 20–80 min)	GC-FPD	TPrP, TnBP, TCEP, TCPP, TPhP, TEHP, and TCrP	73.2–101.8	3.3–7.6	0.7–11.6	[19]

MASE (3 h)	LC-MS/MS	TnBP	100–112	2–13	3 (LOQ)	[15]

DLLME (10 min)	GC-NPD	TnBP, TEHP	TnBP: 94–104 TEHP: 40–114	TnBP: 2–6 TEHP: 9–17	TnBP: 10 TEHP: 80 (LOQ)	[27]

SFO-DLLME (8 mL, 12 min)	LC-MS/MS	TEP, TBP, TCEP, TBEP, TCPP, TTP, TPhP, and TEHP	TEP: 48–58, 86–113 for others	3.2–9.3	20–70	This study

## References

[1] Carlsson H, Nilsson U, Becker G, Östman C (1997). Organophosphate ester flame retardants and plasticizers in the indoor environment: analytical methodology and occurrence. *Environmental Science and Technology*.

[2] Staaf T, Östman C (2005). Organophosphate triesters in indoor environments. *Journal of Environmental Monitoring*.

[3] Fries E, Püttmann W (2001). Occurrence of organophosphate esters in surface water and ground water in Germany. *Journal of Environmental Monitoring*.

[4] Marklund A, Andersson B, Haglund P (2003). Screening of organophosphorus compounds and their distribution in various indoor environments. *Chemosphere*.

[5] Reemtsma T, García-López M, Rodríguez I, Quintana JB, Rodil R (2008). Organophosphorus flame retardants and plasticizers in water and air I: occurrence and fate. *Trends in Analytical Chemistry*.

[6] Ericsson M, Colmsjö A (2003). Dynamic microwave-assisted extraction coupled on-line with solid-phase extraction and large-volume injection gas chromatography: determination of organophosphate esters in air samples. *Analytical Chemistry*.

[7] Wang X, Liu J, Yin Y (2010). The pollution status and research progress on organophosphate ester flame retardants. *Progress in Chemistry*.

[8] Yan XJ, He H, Peng Y (2012). Determination of organophosphorus flame retardants in surface water by solid phase extraction coupled with gas chromatography-mass spectrometry. *Chinese Journal of Analytical Chemistry*.

[9] Martínez-Carballo E, González-Barreiro C, Sitka A, Scharf S, Gans O (2007). Determination of selected organophosphate esters in the aquatic environment of Austria. *Science of the Total Environment*.

[10] Möller A, Sturm R, Xie Z, Cai M, He J, Ebinghaus R (2012). Organophosphorus flame retardants and plasticizers in airborne particles over the Northern Pacific and Indian Ocean toward the polar regions: Evidence for global occurrence. *Environmental Science and Technology*.

[11] Stapleton HM, Klosterhaus S, Eagle S (2009). Detection of organophosphate flame retardants in furniture foam and U.S. house dust. *Environmental Science and Technology*.

[12] Makinen MSE, Makinen MRA, Koistinen JTB (2009). Respiratory and dermal exposure to organophosphorus flame retardants and tetrabromobisphenol A at five work environments. *Environmental Science and Technology*.

[13] Quintana JB, Rodil R, Reemtsma T (2006). Determination of phosphoric acid mono- and diesters in municipal wastewater by solid-phase extraction and ion-pair liquid chromatography-tandem mass spectrometry. *Analytical Chemistry*.

[14] Rodil R, Quintana JB, Reemtsma T (2005). Liquid chromatography-tandem mass spectrometry determination of nonionic organophosphorus flame retardants and plasticizers in wastewater samples. *Analytical Chemistry*.

[15] Quintana JB, Reemtsma T (2006). Potential of membrane-assisted solvent extraction for the determination of phosphoric acid triesters in wastewater samples by liquid chromatography-tandem mass spectrometry. *Journal of Chromatography A*.

[16] García-López M, Rodríguez I, Cela R (2010). Mixed-mode solid-phase extraction followed by liquid chromatography-tandem mass spectrometry for the determination of tri- and di-substituted organophosphorus species in water samples. *Journal of Chromatography A*.

[17] Wang X-W, Liu J-F, Yin Y-G (2011). Development of an ultra-high-performance liquid chromatography-tandem mass spectrometry method for high throughput determination of organophosphorus flame retardants in environmental water. *Journal of Chromatography A*.

[18] Chen D, Letcher RJ, Chu S (2012). Determination of non-halogenated, chlorinated and brominated organophosphate flame retardants in herring gull eggs based on liquid chromatography-tandem quadrupole mass spectrometry. *Journal of Chromatography A*.

[19] Xing YN, Wang X, Chen ZY, Suo YY, Lin HX (2012). Determination of organophosphate esters fire retardant in textile by solid phase extraction combined with GC/MS method. *Chinese Journal of Analytical Chemistry*.

[20] Wang C, Li L, Xie T, Zhang W, Liu C, Zhu N (2011). Simultaneous determination of six organophosphorous flame retardants in textiles by gas chromatography-tandem mass spectrometry combined with microwave assisted extraction. *Chinese Journal of Chromatography*.

[21] Mu J, Li X, Zhang B, Jiang L (2007). Determination of three organophosphorous flame retardants in textiles by gas chromatography. *Chinese Journal of Chromatography*.

[22] Gao QZh, Deng YH, Hu XB, Yang GSh, Sun Ch, He H (2013). Determination of organophosphate esters in water samples using an ionic liquid-based sol-gel fiber for headspace solid-phase microextraction coupled to gas chromatography-flame photometric detector. *Journal of Chromatography A*.

[23] Wang Y-Y, Zhao G-Y, Chang Q-Y, Zang X-H, Wang C, Wang Z (2010). Developments in liquid-phase microextraction method based on solidification of floating organic drop. *Chinese Journal of Analytical Chemistry*.

[24] Ding ZQ, Zhang QY (2010). Dispersive liquid-liquid microextraction based on solidification of floating organic drop combined with high performance liquid chromatography for determination of chlorophenols in aqueous samples. *Chinese Journal of Analytical Chemistry*.

[25] Yang B, Yang HS, Chen F, Hua YL, Jiang YM (2013). Phytochemical analyses of *Ziziphus jujuba* Mill. Var. spinosa seed by ultrahigh performance liquid chromatography-tandem mass spectrometry and gas chromatography-mass spectrometry. *Analyst*.

[26] Rodríguez I, Calvo F, Quintana JB, Rubí E, Rodil R, Cela R (2006). Suitability of solid-phase microextraction for the determination of organophosphate flame retardants and plasticizers in water samples. *Journal of Chromatography A*.

[27] Tsao Y-C, Wang Y-C, Wu S-F, Ding W-H (2011). Microwave-assisted headspace solid-phase microextraction for the rapid determination of organophosphate esters in aqueous samples by gas chromatography-mass spectrometry. *Talanta*.

[28] García-López M, Rodríguez I, Cela R (2007). Development of a dispersive liquid-liquid microextraction method for organophosphorus flame retardants and plastizicers determination in water samples. *Journal of Chromatography A*.

